# Insights into Interactions of Flavanones with Target Human Respiratory Syncytial Virus M_2-1_ Protein from STD-NMR, Fluorescence Spectroscopy, and Computational Simulations

**DOI:** 10.3390/ijms21062241

**Published:** 2020-03-24

**Authors:** Hêmily M. R. Piva, Jéssica M. Sá, Artemiza S. Miranda, Ljubica Tasic, Marcelo A. Fossey, Fátima P. Souza, Ícaro P. Caruso

**Affiliations:** 1 Department of Physics, Instituto de Biociências, Letras e Ciências Exatas (IBILCE), UNESP, São José do Rio Preto 15054-000, Brazil; hemily.mutti@gmail.com (H.M.R.P.); jessica.marostica@unesp.br (J.M.S.); marcelo.fossey@unesp.br (M.A.F.); 2 Multiuser Center for Biomolecular Innovation (CMIB), Instituto de Biociências, Letras e Ciências Exatas (IBILCE), UNESP, São José do Rio Preto 15054-000, Brazil; artemizammiranda@hotmail.com; 3 Department of Biology, Instituto de Biociências, Letras e Ciências Exatas (IBILCE), UNESP, São José do Rio Preto 15054-000, Brazil; 4 Organic Chemistry Department, Institute of Chemistry, UNICAMP, Campinas 13083-970, Brazil; ljubica@unicamp.br; 5 National Center for Nuclear Magnetic Resonance of Macromolecules, Institute of Medical Biochemistry and National Center for Structure Biology and Bioimaging (CENABIO), UFRJ, Ilha do Fundão, Rio de Janeiro 21941-902, Brazil

**Keywords:** flavanones, hRSV M2-1, STD-NMR, fluorescence spectroscopy, molecular docking, molecular dynamics

## Abstract

The human Respiratory Syncytial Virus (hRSV) is the most frequent agent of respiratory infections in infants and children with no currently approved vaccine. The M_2-1_ protein is an important transcriptional antitermination factor and a potential target for viral replication inhibitor development. Hesperetin (HST) and hesperidin (HSD) are flavonoids from the flavanone group, naturally found in citrus and have, as one of their properties, antiviral activity. The present study reports on the interactions between hRSV M_2-1_ and these flavanones using experimental techniques in association with computational tools. STD-NMR results showed that HST and HSD bind to M_2-1_ by positioning their aromatic rings into the target protein binding site. Fluorescence quenching measurements revealed that HST had an interaction affinity greater than HSD towards M_2-1_. The thermodynamic analysis suggested that hydrogen bonds and van der Waals interactions are important for the molecular stabilization of the complexes. Computational simulations corroborated with the experimental results and indicated that the possible interaction region for the flavonoids is the AMP-binding site in M_2-1_. Therefore, these results point that HST and HSD bind stably to a critical region in M_2-1_, which is vital for its biological function, and thus might play a possible role antiviral against hRSV.

## 1. Introduction

The human respiratory syncytial virus (hRSV) is the leading cause of lower respiratory tract infections in infants and children worldwide [1,2] and is also responsible for the morbidity and mortality of the elderly and immunocompromised patients [3,4]. It belongs to the genus *Orthopneumovirus*, family Pneumoviridae and order Mononegavirales that consists of enveloped viruses [5]. The hRSV genome is composed of simple and nonsegmented negative-sense RNA that codes to eleven key proteins. Among those, two proteins are encoded by the M_2_ gene and translated from overlapping reading frames, which are named M_2-1_ and M_2-2_ proteins [6].

The hRSV M_2-1_ protein is an essential transcription factor of the viral RNA-dependent RNA polymerase (RdRp) complex that permits RdRp to reach the end of each transcriptional unit [7,8] and to complete the transcription [9]. Evidence suggests that M_2-1_ is located between the ribonucleoprotein complex and the M protein [10] and that its association with the replisome increases the replication activity of viral RNA [11]. The M_2-1_ monomer has 194 residues of amino acids mainly arranged in α-helices and this protein forms a stable tetramer in solution [12]. The M_2-1_ structure is predicted to comprise four functionally distinct regions: the N-terminal region linked to a zinc-finger domain [13]; the α-helix region responsible to mediate the oligomerization [12]; the globular domain capable of binding to the phosphoprotein protein and RNA; and non-structured C-terminal region [14].

Currently, there is no effective drug or available vaccine for hRSV. The usual health care facilities commonly offer to treat symptoms and count on oxygen support and hydration, but are not effective, have great costs and involve high mortality rates [15,16]_._ There are some therapies developed to treat viral-mediated diseases, for example, the application of humanized monoclonal antibody Palivizumab that binds to the fusion (F) protein and prevents host cell entry of the virus [17]; or the antiviral therapy with Ribavirin which is a guanine nucleotide analog [18]. However, these treatments have high costs and need long-term therapies, show substantial side effects, and low efficacy against the hRSV [18,19,20,21].

Nature is a potentially rich source of antiviral agents, where flavonoids occupy the lead as interesting compounds to be tested against many viral infections [22,23,24]. Hesperetin (HST) and its glycosylated form hesperidin (HSD) are flavanones mainly found in citrus species, which among many pharmacological properties show antiviral effects [25,26,27]. Kaul et al. (1985) showed that HST can reduce the intracellular replication of herpes simplex virus type 1 (HSV-1), poliovirus type 1, parainfluenza virus type 3 (Pf-3), and respiratory syncytial virus [28]. In particular, HST presented an anti-replicative activity of 88% against RSV in plaque reduction assays. A Chinese herbal formula named Jiawei-Yupingfeng-Tang (JYT) has in its formulation 11.1% (w/w) of hesperidin, and is used to prevent respiratory infections caused by influenza virus and hRSV, as well [29].

M_2-1_ can be considered as the target protein to the potential antiviral activity of small molecules, such as flavonoids. Previous studies showed for example that the quercetin [30] and with its acetylated derivatives [31] can bind to the antitermination factor M_2-1_. Therefore, the present work aimed to investigate the binding interaction of HST and HSD with the hRSV M_2-1_ protein by using experimental techniques of STD-NMR and fluorescence spectroscopy in association with computational tools of molecular docking and molecular dynamics. Such characterization at molecular level provides details of this M_2-1_/flavanone binding and thus sheds light on the possible development of a new strategy against hRSV.

## 2. Results and Discussion

### 2.1. Production of the Recombinant hRSV M_2-1_ Protein

**Figure 1:**
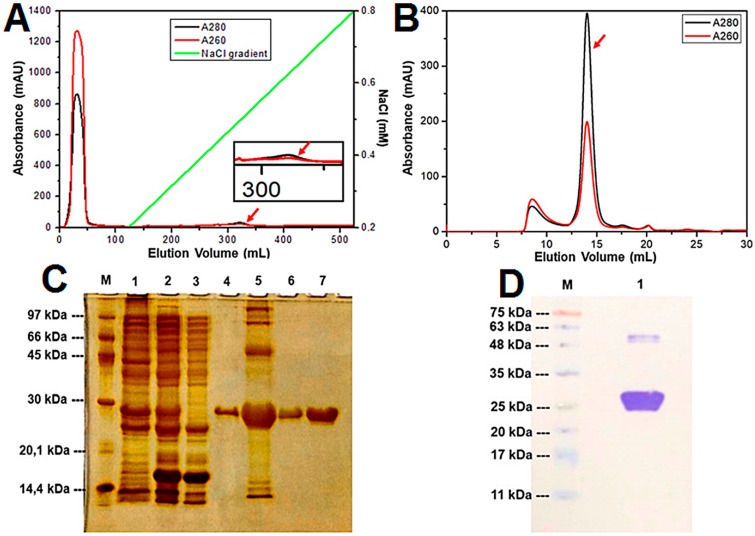
Purification of the recombinant hRSV M_2-1_ protein. (**A**) Sample containing M_2-1_ protein was loaded onto a HiPrep 16/10 Heparin FF affinity column and eluted at 465–540 mM NaCl in a 0.2 to 1.0 M NaCl gradient. (**B**) The concentrated protein fractions from affinity chromatography were loaded onto a Superdex 200 10/300 gel filtration column between 0.65–0.85 column volume (CV) and RNA-free (A_260_/A_280_ < 1.0). The red arrows, in each step of the purification, indicate the elution moment of the protein of interest. (**C**) SDS-PAGE analysis followed by silver nitrate staining of M_2-1_ protein at different purification stages. M: molecular mass marker; 1: sample after precipitation with ammonium sulfate; 2: sample after 4 h of RNAse treatment; 3: flow of affinity column; 4: M_2-1_ eluted with 32% of buffer B in a 0.2 to 1.0 M NaCl gradient in the affinity column; 5: fractions eluted from the affinity column and concentrated in 10 kDa cutoff concentrator; 6: M_2-1_ eluted in the exclusion chromatography; 7: M_2-1_ eluted in the exclusion chromatography and concentrated. (**D**) Immunodectetion (Western Blotting gel) of the recombinant hRSV M_2-1_ protein. M: molecular weight marker; 1: fraction eluted after molecular exclusion chromatography. The molecular mass of the M_2-1_ protein, predicted by ProtParam tool from ExPASy server, is approximately 25 kDa.

The highest level of M_2-1_ expression was obtained with 0.3 mM IPTG and 50 µM ZnSO_4_ for 15 h at 28 °C. The lysed cell extract was first purified using a Hiprep 16/10 Heparin FF affinity column (GE HealthCare) and the M_2-1_ protein was eluted at 465–540 mM NaCl in a salt gradient (Figure 1A). Next, the fractions from the peak containing the interest protein were concentrated and injected onto a size exclusion column Superdex 200 10/300 (GE HealthCare, Chicago, IL, USA) for additional purification and for exchanging the buffer solution, 50 mM K_2_HPO_4_/KH_2_PO_4_ (pH 7.4) containing 150 mM NaCl and 1 mM DTT. In this step, the purified M_2-1_ protein was eluted in the peak volume fractions 13–17 mL (Figure 1B). SDS-PAGE followed by silver nitrate staining monitored all steps of purification and revealed the presence of a band at approximately 25 kDa corresponding to the M_2-1_ protein (Figure 1C). This band visualized in the 15% SDS-PAGE was confirmed as hRSV M_2-1_ protein by Western Blotting (immunodetection assay, Figure 1D). In the end of the production and purification process, it was obtained ~10 mg·mL^−1^ of pure M_2-1_ (A_260_/A_280_ < 1.0) per liter of expression for performing of the flavonoid-binding experiments.

### 2.2. STD-NMR for Investigating the M_2-1_/Flavanone Binding

**Figure 2:**
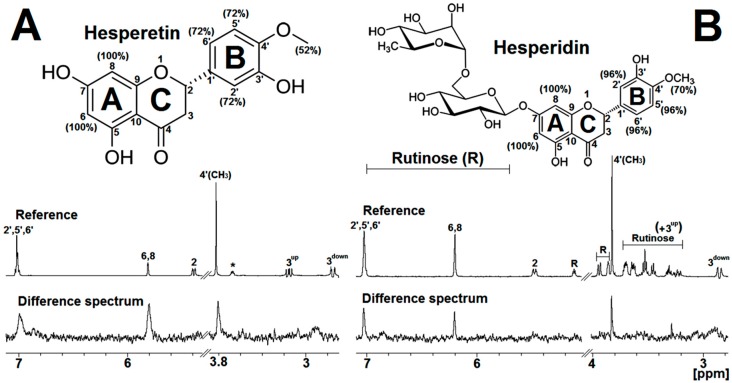
STD-NMR results obtained for the M_2-1_/flavanone binding collected at 600 MHz and 298 K (25 °C). (**A**) At the top, it is presented the molecular structure of the HST with percentage of the binding epitopes in parentheses. In the middle, 1D ^1^H NMR reference spectrum assigned with respective hydrogen atoms named in the molecular structure. At the bottom, 1D ^1^H NMR difference spectrum for the binding of hesperetin (HST) to M_2-1_ protein. The asterisk denotes a sign of ethanol. (**B**) On the top, it is shown the molecular structure of the hesperidin (HSD) with percentage of the binding epitopes in parentheses. In the middle, 1D ^1^H NMR reference spectrum assigned with respective hydrogen atoms named in the molecular structure. The signal from the rutinose group of HSD are depicted by R letter. At the bottom, 1D ^1^H NMR difference spectrum for the binding of HSD to M_2-1_ protein. The NMR spectra were acquired with 50 µM of protein and 250 µM of HST or HSD. For better visualization of the NMR data, the spectra were expanded to the spectral region with well-resolved difference signals. The signal region from water (HDO) molecule was removed.

In this study, STD-NMR technique was used to investigate the interaction between the hRSV M_2-1_ protein and the flavonoids hesperetin (HST) and hesperidin (HSD). From the difference spectrum in Figure 2, it is possible to note the respective binding epitopes of HST or HSD with the M_2-1_ protein. The difference spectrum for the M_2-1_/HST binding (bottom, Figure 2A) reveals the presence of the resonance peaks at 7.00 and 5.80 ppm corresponding to the ^1^H protons on the aromatic rings B (hydrogens: 2′, 5′, and 6′) and A (hydrogens: 6 and 8), respectively, and also the resonance at 3.80 ppm regarding the methyl group (hydrogens of 4′CH_3_) attached to the B-ring of the flavonoid. The map of binding epitopes of the HST (top, Figure 2A) shows that the hydrogens on the A-ring of this flavonoid received more saturation transfer (denoted by 100%) from the protein and, therefore, these ^1^H protons present the highest STD effect for the interaction with M_2-1_. This result suggests that the A-ring of the HST is more buried inside the binding site in the hRSV M_2-1_. In case of the HSD, the difference spectrum of its interaction with M_2-1_ presents resonance peaks at 7.00, 6.20, and 3.80 ppm corresponding to hydrogens on the B-ring, A-ring, and methyl group attached to B-ring, respectively (bottom, Figure 2B). The binding epitopes of the HSD (top, Figure 2B) reveal that hydrogens on the A-ring of the flavonoid received more saturation transfer from the hRSV M_2-1_, just as it happens for the HST. However, it is noteworthy that the STD effect for the ^1^H proton on the B-ring exhibits a significant contribution of 96%, which is higher than that observed for HST B-ring (72%). The hydrogens from glycosylation of the HSD present no STD effect under interaction with the protein, which indicates that the rutinose group of this flavonoid is exposed to the solvent.

Therefore, the outcomes from STD-NMR experiments suggest that the aromatic rings A and B of the HST and HSD play a key role in the interaction with the hRSV M_2-1_ protein, mainly the A-ring. The rutinose group attached to the A-ring of the HSD likely does not interact significantly with binding site in the protein, but this glycosylation may affect the behavior of the binding of HSD to M_2-1_. The identification of the binding epitopes for HST and HSD provides pivotal information for molecular modeling experiments, making it possible to identify and refine possible sites of these molecules as well as to search for their binding sites in the hRSV M_2-1_ protein.

### 2.3. Fluorescence Quenching of M_2-1_ by Hesperetin and Hesperidin

**Figure 3:**
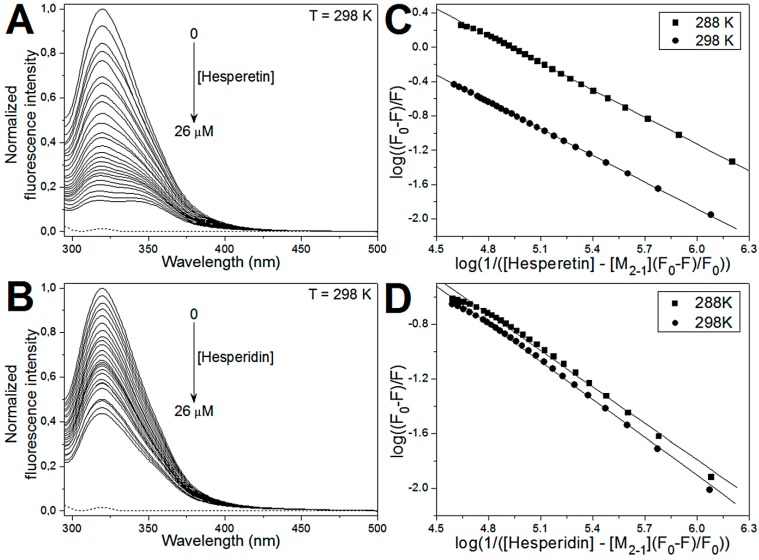
Emission spectra of human Respiratory Syncytial Virus (hRSV) M_2-1_ protein in the absence and presence of (**A**) HST and (**B**) HSD at 298 K (25 °C). The concentration of M_2-1_ protein was 6 µM and the concentration of the flavanones varied from 0 to 26 µM. The dotted line represents the buffer emission spectrum. The double-log plots for the interaction of the (**C**) HST and (**D**) HSD with the M_2-1_ protein. The fluorescence experiments performed at 288 K are shown as squares and at 298 K are depicted as circles. The solid line represents the linear fitting.

The fluorescence quenching experiments were used to investigate the interaction between the flavonoids in the study and the M_2-1_ protein. Figure 3 shows the fluorescence quenching spectra of the protein in the presence of the HST (Figure 3A) and HSD (Figure 3B) at 298 K (25 °C). It is possible to observe that M_2-1_ fluorescence intensity decreases with increasing concentration of HST or HSD, indicating that these flavanones affect the molecular nanoenvironment of the fluorophores in the protein.

From the analysis of the fluorescence quenching data using double-log plot (Figure 3C and 3D), the number of ligands per protein (*n*) and the binding constant (K_b_) can be determined for the interaction between the flavonoids and the M_2-1_ protein. The results of the linear fitting showed that the binding constants (K_b_) obtained for the M_2-1_/flavanones complex are in order of 10^4^ M^−1^, and the K_b_ values of the M_2-1_/HST interaction are up to eight-fold higher than those for the binding of the HSD to the protein. This difference may be due to glycosylation that can cause a reorientation on the binding mode of hesperidin and probably promote a decrease in its affinity toward the protein. Similar results were reported to the interaction between two aglycone flavonoids (baicalein and quercetin) and their glycosides (baicalin and quercitrin) with serum albumin, in which the glycosylation decreased the binding affinity with protein [32]. In addition, the binding stoichiometry revealed that a single site takes place to the interaction of the HST and HSD with the monomeric unit of hRSV M_2-1_ protein.

The K_b_ obtained in the two different temperatures (288 and 298 K) was used to characterize the non-covalent interactions involved in the binding of the flavonoids to the M_2-1_ protein. The K_b_ values were employed into the Van’t Hoff equation (Eq. (3)) to calculate the binding enthalpy change (ΔH) for the investigated temperature range. The values of Gibbs free energy (ΔG) and entropy (ΔS) changes were obtained from Eqs. (4) and (5), respectively, and along with the ΔH values are shown in Table 1. It is possible to note from the thermodynamic parameters analysis that the binding process of HST or HSD to hRSV M_2-1_ protein is spontaneous (ΔG < 0) and exothermic (ΔH < 0), indicating enthalpically favorable processes. For the interaction with the HST, the entropy change contributes unfavorably (ΔS < 0) to ΔG with the binding reaction being enthalpically driven. On the other hand, the entropic term contributes favorably (ΔS > 0) to ΔG for the interaction with the HSD, resulting in an entropically driven binding reaction. In the case of the M_2-1_/HST binding with ΔH < 0 and ΔS < 0, the stabilization of the complex is driven by hydrogen bonds and van der Waals interactions [33]. The thermodynamic parameters of the M_2-1_/HSD binding (ΔH < 0 and ΔS > 0) doesn’t clearly suggest which are the non-covalent interaction that stabilizes the complex; however, due to the structural similarity of both flavanones, it is worth to take into account that hydrogen bonds and van der Waals interactions performed by the phenolic groups of HSD (as for HST) are probably important contributions to the complex stabilization. In this specific case, the ΔS > 0 may be interpreted as a release of ordered water molecules (desolvation) from the binding regions of the HSD in the protein [34], likely promoted due to the solvent exposure of the rutinose (glycosylation) group of the flavonoid.

### 2.4. Computational Approach of the hRSV M_2-1_/flavonoid Complexes

**Figure 4:**
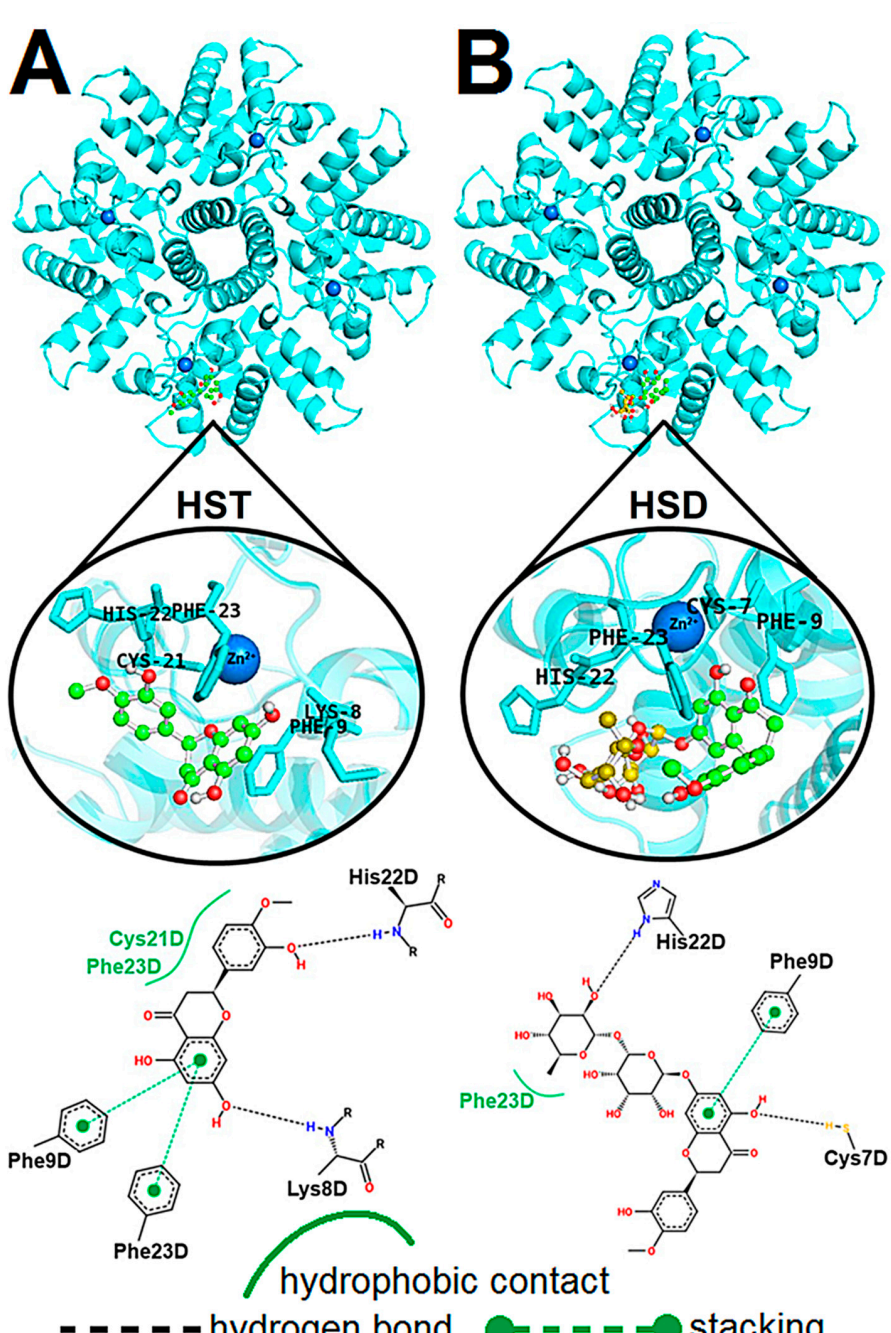
Possible localization of the flavonoids (**A**) HST and (**B**) HSD in the AMP-binding site of the hRSV M_2-1_ protein calculated by molecular docking. At the top, the structural models of the complexes with the lowest energy conformers from the largest cluster of the docking calculations. The protein is denoted by cartoon, the zinc atoms as blue spheres, and flavonoids as sticks and balls model (C: green; O: red, H: white; C from rutinose are in yellow for HSD). In the middle, three-dimensional structural details of the binding nanoenvironment of flavonoids, highlighting the main residues of the M_2-1_ protein involved in the non-covalent interactions. At the bottom, the two-dimensional representation of the interaction maps between the flavanones and residues of the M_2-1_ protein. The hydrogen bonds are denoted by dotted line, stacking interaction by dotted green line, and hydrophobic contact by solid green line. The letter D at the end of the residue names depicts the tetramer chain. The structural representations were made in PyMol and the interaction maps were calculated using PoseView program.

The structural models of the hRSV M_2-1_/flavonoid complexes were calculated by using the AutoDock 4.2 program [35] as mentioned in Section 3.7. Figure 4 shows the structural models of the M_2-1_/flavonoid complexes determined from the docking calculations. The poses of HST and HSD correspond to the lowest energy conformers from the most populated cluster. The lowest energy conformer of HST presented a docking score of −4.16, which is better ranked than the score value observed for HSD, −2.59.

Figure 4A shows the structural model for the interaction of the HST with the M_2-1_. The binding of the flavonoid takes place in a cavity formed by the zinc-finger domain from a monomer (chain D) of the tetramer and the core domain from another one (chain A) (top, Figure 4A). An analysis of the molecular nanoenvironment of the binding site for HST in M_2-1_ reveals that the closest amino acid residues interacting with the flavonoid are: Lys8, Phe9, Cys21, His22, and Phe23 (middle, Figure 4A), which are all from chain D of the hRSV M_2-1_ tetramer. The PoseView program [36] was used to map the non-covalent interactions formed between these residues and HST (bottom, Figure 4A). His22 and Lys8 establish hydrogen bonds with hydroxyl groups of the HST, while Cys21 and Phe23 participate in hydrophobic contacts and the side chain of Phe9 and Phe23 form stacking interactions with the A-ring of the HST. These non-covalent interactions identified for the M_2-1_/hesperetin complex are in agreement with the analysis of the thermodynamic parameters (Section 2.3). The position of the A-ring between the side chains of Phe9 and Phe23 along with stabilization performed by hydrogen bond with Lys8 also corroborate with the outcomes from the STD-NMR experiments (Section 2.2), which highlighted this benzene group as the most buried inside the hRSV M_2-1_ binding site.

For the M_2-1_/HSD complex (Figure 4B), the conformer of HSD is located in the same cavity as HST; however, it presents a different orientation of its aromatic rings A and B in the protein binding site when compared to the pose of HST. This different orientation can be due to the presence of the rutinose group in the HSD molecular structure, which is positioned to expose glycosylation to the solvent (carbon atoms in yellow, Figure 4B). The binding nanoenvironment for the HSD in the protein shows that the closest amino acid residues (from chain D) interacting with the conformer are: Cys7, Lys8, Phe9, His22, and Phe23 (middle, Figure 4B), which are similar to those presented for the M_2-1_/HST complex. An analysis of the non-covalent interactions between these residues and the HSD conformer reveal the occurrence of hydrogen bonds and stacking interaction for the complex stabilization, similar to observed for HST (bottom, Figure 4B). Hydroxyl groups on rutinose and A-ring of the HSD form hydrogen bonds with His22 and Cys7, respectively, while Phe9 participates in a stacking interaction with the A-ring and Phe23 establishes a hydrophobic contact with the methyl group on the HSD rutinose. These computational results are in agreement with the analysis of the HSD binding epitopes determined by STD-NMR experiments and also suggest that the rutinose group promotes an orientation of the HSD phenolic region different from HST inside the binding site in M_2-1_, what may explain the smaller affinity of the HSD to the protein when compared to HST.

The docking results indicate that Cys7, Lys8, Phe9, His22, and Phe23 play a key role in the binding of the HST and HSD to hRSV M_2-1_ since these are involved in the formation of hydrogen bonds and stacking interactions with the flavanones. Similar results were found by Guimarães et al. (2018) for the complex formed between the M_2-1_ protein and acetylated quercetin derivatives [31]. In this study, Lys8 and Phe23 were highlighted as important residues for the binding of the acetylated flavonoids. Leyrat et al. (2014) also showed via X-ray crystallography that the adenine moiety of AMP interacts with the zinc-finger domain of the M_2-1_ protein mainly via stacking interaction with Phe23 and hydrogen bond with the backbone nitrogen of Lys8. Therefore, the analysis of the docking calculations shows that the HST and HSD can interact in the M_2-1_/RNA binding region, known to be vital for the biological function of this protein [37].

**Figure 5:**
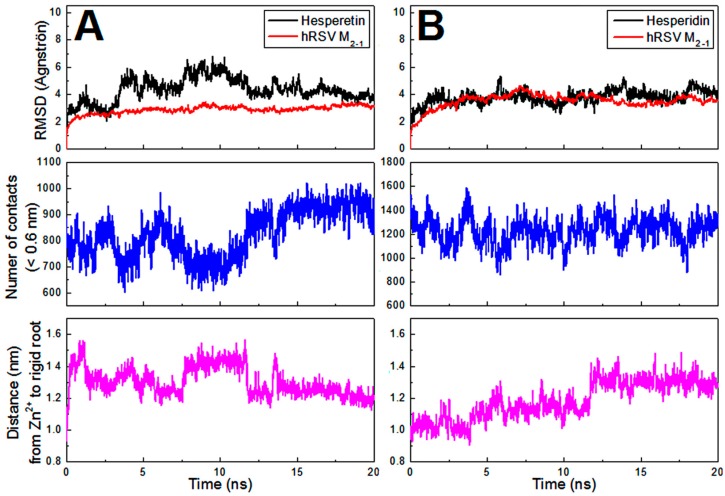
Analysis of the parameters from 20 ns molecular dynamics simulations of the structural models of the complexes formed with the HST (**A**) and HSD (**B**) determined by AutoDock program. On the top, the root mean square deviations (RMSDs) of non-hydrogen atoms of the flavonoids (black line) and backbone atoms of the M_2-1_ protein (red line) from the starting structure (AutoDock model) for the complexes are reported as a function of time. In the middle, number of contacts between atoms of the M_2-1_ and flavanones for distances < 0.6 nm. At the bottom, distance from Zn^2+^ atom in the zinc-finger domain of the protein to the rigid root of the flavanones. As molecular dynamics simulations were performed in duplicate, the mentioned parameters were presented as average.

The molecular dynamics (MD) calculations were performed to check the stability of the structural models of the M_2-1_/flavonoid complexes generated from the AutoDock 4.2 program [35]. The parameters analyzed (see Section 3.8) from the 20 ns MD simulations for the complexes formed with the HST and HSD are presented as average values for two independent calculations in Figure 5A,B, respectively. The non-averaged values of these parameters are shown in Figure S1 in Supplemental Material. The RMSDs of non-hydrogen atoms of the flavonoids and backbone atoms of the M_2-1_ protein from the starting structure (AutoDock model) for the complexes are reported as a function of time in the Figure 5 topper part. Stable RMSD values of approximately 4.6 and 3.9 Å (on average) for HST and HSD in complex with M_2-1_ were reached after 2 and 3 ns of simulation, respectively. This result points out that HST and HSD established equilibrium positions relatively similar to those in their starting structural models (Figure S2). As an internal control of the MD simulations, the values of RMSD for the backbone atoms of the protein were also calculated. These values presented no significant fluctuation all over the simulations (~3 Å on average) and therefore indicate that the protein structure remained stable. Another control calculation was performed by extending to 80 ns one of the two MD simulations for each flavonoid. Figure S3 reveals that even for a longer simulation, the RMSD values remain stable and thus reinforce the binding stability of the structural models of the complexes. The evaluation of the number of contacts between atoms of M_2-1_ and flavonoids for distances <0.6 nm (middle, Figure 5) shows that HST and HSD were interacting with the protein throughout the 20 ns MD simulations. Steady values of distance (bottom, Figure 5) from Zn^2+^ ion of the zinc-finger domain to the rigid root of the flavanones (for details see Section 3.8) corroborate with the analyses of the number of contacts, since these distances of ~1.25 nm (on average) reveal that both polyphenolic compounds remained at the binding site in M_2-1_ initially determined from the docking calculations and thus were in contact with the protein all over the simulation time.

The Molecular Mechanics Poisson-Boltzmann Surface Area (MM-PBSA) [38,39] was used to provide an accurate assessment of the theoretical binding free energies (ΔG*_binding_*) for the interaction between the flavanones and M_2-1_. Table 2 shows the values of ΔG*_binding_* calculated from the last 10 ns of the MD trajectories for the structural models of the M_2-1_/flavonoid complexes, as well as their corresponding average values. It is possible to observe from Table 2 that, on average, the value of ΔG*_binding_* for HST (−35.2 kcal.mol^−1^) was larger in magnitude than that determined for HSD (−28.2 kcal.mol^−1^), indicating that HST has a higher affinity to M_2-1_ when compared to HSD. This result corroborates with the experimental affinity tendency obtained from the fluorescence quenching data, which showed that the M_2-1_/HST complex presents binding constant values higher than those for the complex with HSD.

The accumulated percentage occupancies of hydrogen bonds formed between M_2-1_ and flavonoids during MD trajectory were obtained for all simulations and are shown in Table 3 and Table 4 for HST and HSD, respectively. The amino acid residues of the protein involved in hydrogen bonds with average values of accumulated percentage occupancy >10% were considered as a significant non-covalent interaction for the molecular stabilization of the M_2-1_/flavonoid complex. For the M_2-1_/HST binding, backbone atoms of the residues Lys8 and Arg20 from chain D showed occupancy of 70.3% and 14.6%, respectively; while side chain atoms of Lys8 and Asn17 from chain D and Arg169 and Asn174 from chain A presented occupancy of 25.8%, 26.8%, 13.9%, and 69.8%, respectively (Table 3). For the M_2-1_/HSD complex, HSD forms hydrogen bonds with backbone atoms of the residues Lys8, Phe9, and Arg20 from chain D showing occupancy of 14.8%, 10.5%, and 22.6%, respectively, and with a side chain atom of His22 from chain D presenting occupancy of 17% (Table 4). The larger number of hydrogen bonds with the highest frequencies of occurrence for the M_2-1_/HST complex all over the MD simulations follows the experimental results and MM-PBSA calculations which suggest a higher affinity of this polyphenol regarding HSD, considering that hydrogen bonds play a key role in stabilization of the protein/flavonoid binding. As noted for docking calculations, the residue Lys8 and Arg20 stand out in the formation of hydrogen bonds with the flavonoids hesperetin and its glycosylated form hesperidin inside the AMP-binding site in hRSV M_2-1_ protein.

## 3. Material and Methods

### 3.1. Material

Hesperetin (HST), kanamycin sulfate, chloramphenicol, D-sorbitol, β-mercaptoethanol (BME), isopropyl β-D-1-thiogalactopyranoside (IPTG), ethylenediamine tetraacetic acid (EDTA), dimethyl sulfoxide (DMSO), deuterated DMSO-d_6_, deuterium oxide (^2^H_2_O and D_2_O) ribonuclease A, Tris-HCl, NaCl, ZnSO_4_, (NH_4_)_2_SO_4_, K_2_HPO_4_, and KH_2_PO_4_ were purchased from Sigma-Aldrich (Saint Louis, MO, USA); 1,4-dithiothreitol (DTT) from InLab; and Amicon Ultra-15 centrifugal filter (MWCO: 3.0 kDa) from Millipore. Hesperidin (HSD) was produced, purified [40] and kindly provided by Prof. Dr. Ljubica Tasic from University of Campinas (UNICAMP), Brazil.

### 3.2. Expression and Purification of the hRSV M_2-1_ Protein

The optimization of codons of the M_2-1_ gene and the construction of the recombinant plasmid was carried out by ATUM Company (Newark, CA, USA). A M_2-1_ cDNA from hRSV A2 strain was inserted into pD441-NHT vector, allowing the expression of the full-length M_2-1_ with N-terminal fused to a His_6_-tag. The resulting plasmid was transformed in *Escherichia coli* BL21 RIL (Agilent Technologies), which a single colony growing until reached optical density at 600 nm (OD_600_) of 0.6 at 37 °C. The large scale production of the protein was performed in Luria-Bertani (LB) medium containing 500 mM D-sorbitol, 50 µg/mL kanamycin sulfate and 34 µg/mL chloramphenicol, and induction was made with 0.3 mM IPTG with 50 µM of ZnSO_4_ to aid the correct folding of the zinc-finger domain of the protein, for 15 h at 28 °C. The purification was performed as previously described [41] with some modifications. Cells were recovered by centrifugation and suspended in 40 mL of lysis buffer [100 mM Tris-HCl (pH 8.0), 0.6 M NaCl; 5 mM β-mercaptoethanol (BME), 1 mM EDTA] per 1 L of pelleted culture. Next, the cells were lysed by sonication and centrifuged for 30 min at 15,000× *g* at 4 °C. The clarified supernatant containing the M_2-1_ protein was precipitated via addition of solid ammonium sulfate to 40% saturation. The precipitated protein was collected by centrifugation, resuspended, and dialyzed against buffer A [50 mM Tris-HCl (pH 7.0), 0.2 M NaCl, 1 mM BME). After dialysis, the sample containing the interest protein was treated with 1 mg/L of ribonuclease A and incubated for 4 h at 37 °C. After this time, the sample was centrifuged at 15,000× *g* for 30 min at 4 °C and the soluble fraction was subjected to a Hiprep 16/10 Heparin FF affinity column (GE HealthCare) previously equilibrated with buffer A. The column was washed with 5 column volumes of buffer A, and M_2-1_-bound protein was eluted with a linear gradient from 0.2 to 1 M NaCl [Buffer B: 50 mM Tris-HCl (pH 7.0), 1 M NaCl, 1 mM BME). The fractions corresponding to the interest protein was concentrated and applied on a Superdex 200 10/300 molecular exclusion chromatography column (GE Healthcare, Chicago, IL, USA) equilibrated with 50 mM K_2_HPO_4_/KH_2_PO_4_ (pH 7.4), 150 mM NaCl, and 1 mM DTT. An isocratic elution with a unit of total column volume and UV monitoring at 260 and 280 nm was carried out. The fractions of pure M_2-1_ protein, with 260/280 absorbance ratio (A_260_/A_280_) less than 1 unit, were pooled and concentrated using Amicon Ultra-15 centrifugal filter (MWCO: 3.0 kDa). The sample purity and detection were assessed by 15% SDS-PAGE and Western Blotting gels, respectively. For immunodetection, the monoclonal anti-polyhistidine primary antibody (Sigma Aldrich, Saint Louis, MO, USA) and polyclonal anti-mouse IgG (Fab specific)-peroxidase secondary antibody were used.

### 3.3. Sample Preparation

The protein concentration was determined spectroscopically (UV-Vis Spectrophotometer, BioMATE 3S, Thermo Scientific, Waltham, MA, USA) using the molar extinction coefficient of 13,200 M^−1^·cm^−1^ per monomeric unit at 280 nm [42]. The stock solutions of hesperetin (302.3 g/mol) and hesperidin (610.6 g/mol) were prepared in DMSO (and deuterated DMSO-d_6_ for NMR experiments) at final concentrations of 1.8 mM. For the flavonoids, the following molar extinction coefficients were used: hesperetin $\varepsilon_{290}$ = (33.2 ± 0.3) × 10^3^ M^−1^cm^−1^ at 290 nm and hesperidin $\varepsilon_{285}$ = (18.1 ± 0.4) × 10^3^ M^−1^cm^−1^ at 285 nm, which were determined spectroscopically.

### 3.4. Saturation Transfer Difference (STD) by Nuclear Magnetic Resonance (NMR)

STD-NMR experiments were performed on a Bruker Avance III 600.13 MHz equipped with a triple resonance 5 mm cryoprobe, having the pulse field gradient along the Z axis. All data were analyzed with Bruker Topspin v3.5. A total concentration of 50 μM hRSV M_2-1_ protein in 50 mM K_2_HPO_4_/KH_2_PO_4_ buffer (pH 7.4) containing 150 mM NaCl and 1 mM DTT at 80/20% H_2_O/D_2_O was used for all measurements. The concentration of the flavonoids used in binding experiments was of 250 μM. The on- and off-resonance frequencies were set at −0.5 and 40 ppm, providing the best profile of protein saturation and STD effect. The saturation time was reached at 3 s. A total of 16 scans with 32 loop counters were collected with 4 dummy scans. For the suppression of the protein signals, it was applied a spinlock filter of 30 ms with 0.776 W. All measurements were performed at 298 K (25 °C) and it was used a flavonoid stock solution solubilized in deuterated DMSO-d_6_.

The STD effect ($I_{STD}$) on a given proton of the ligand was calculated according to the following equation [43,44]:

(1) $$I_{STD}=\frac{I_{off\text{-}resonance}-I_{on\text{-}resonance}}{I{\;}_{off\text{-}resonance}}$$

where *I_on-resonance_* and *I_off-resonance_* are the intensities of the ligand signals in the on- and off-resonance spectrum, respectively. The proton of the flavonoid with the highest STD effect (magnetization transfer maximum) was equal to 100% and the others protons were normalized according to this signal. The identification of ^1^H NMR signals of the flavonoids at buffer solution [50 mM K_2_HPO_4_/KH_2_PO_4_ buffer (pH 7.4) at 80/20% H_2_O/D_2_O containing 150 mM NaCl and 1 mM DTT] was assisted by the resonance assignment from Chemical Book database (www.chamicalbook.com) under access code 520-33-2.

### 3.5. Fluorescence Spectroscopy

The fluorescence measurements were performed on a steady-state ISS-PC1 spectrofluorimeter with temperature control (thermal bath Neslab RTE-221) and equipped with quartz cuvette of 10 mm optical path length. The excitation and emission bandwidths were set at 4 and 8 nm, respectively. The excitation wavelength was of 290 nm and the emission spectra were reported between 295 and 500 nm with 10 accumulations for each collected point. The emission spectrum was corrected for the background fluorescence from the buffer solution and for inner filter effect [45] promoted by the flavonoids. The protein-ligand interaction measures were performed by collecting the fluorescence spectra of the protein in the absence of ligand (2 mL at 6 μM) and checking for quenching of intensities after addition of increasing concentrations of flavonoids, at 288 and 298 K (15 and 25 °C). The flavonoids’ concentrations varied from 0 to 26 μM in the final solution, with the emission of the hRSV M_2-1_ protein analyzed at 320 nm.

For the data analysis, it was hypothesized that the binding sites of the flavonoids on the M_2-1_ protein are equal and independent. Thus, the binding constant (K_b_) and the number of binding sites (*n*) can be calculated using the following equation [46]:

(2) $$\log\left(\frac{F_{0}-F}{F}\right)=n\cdot \log K_{b}+n\cdot \log\frac{1}{\left(\left[L_{T}\right]-\left(\frac{F_{0}-F}{F_{0}}\right)\left[P_{T}\right]\right)}$$

where F_0_ and F are the fluorescence intensity of the M_2-1_ protein in the absence and in the presence of the flavonoids, respectively. [P_T_] and [L_T_] is the total concentration of M_2-1_ protein and ligand (flavonoids), respectively. The values of K_b_ and n for the M_2-1_/flavonoids complex are obtained from the ordinate and slope of the linear fitting from the double-log plot log((F_0_ − F)/F) & log(1/([L_T_] − ((F_0_ − F)/F_0_)[P_T_])), respectively.

### 3.6. Thermodynamic Analysis

In order to investigate the driving forces responsible for the binding of the flavonoids hesperetin and hesperidin to the M_2-1_ protein, the enthalpy change (ΔH) of this interaction was calculated from the Van’t Hoff equation:

(3) $$\ln\frac{K_{b2}}{K_{b1}}=-\frac{\Delta H}{R}\left(\frac{1}{T_{2}}-\frac{1}{T_{1}}\right)$$

where K_b1_ and K_b2_ are binding constants at the absolute temperatures *T*_1_ and *T*_2_, which were 288 and 298 K (15 and 25 °C), respectively. R corresponds to the universal gas constant (1.987 J·K^−1^·mol^−1^). The Gibbs free energy (Δ*G*) and entropy changes (Δ*S*) for the M_2-1_/flavonoids complex were calculated from the following equations:

(4) $$\Delta G=-R\cdot T\cdot \ln K_{b}$$

(5) $$\Delta S=\frac{\Delta H-\Delta G}{T}$$

### 3.7. Molecular Docking

The three-dimensional (3D) crystal structure of hRSV M_2-1_ tetramer was downloaded from the Protein Data Bank under access code 4C3D [14], with some loop regions absent in this structure rebuilt by using Swiss-Model server [47]. 3D structures of the hesperetin and its glycosylated form hesperidin were obtained by structural optimization calculations from the semi-empirical PM6 method using the Gaussian 09 program [48]. The AutoDockTools software [49] was used to prepare M_2-1_ and flavonoids by merging non-polar hydrogen atoms, adding partial charges and atom types. The rigid root of the ligands was generated automatically by setting all possible rotatable bonds and torsions by defining them as active for the compounds. HST and HSD have 5 and 14 rotatable torsions, respectively. The grid maps were generated with 0.375 Å spacing and dimensions of 70 × 70 × 70 points by the AutoGrid 4.2 program [35], which were centered on the binding site of adenosine monophosphate (AMP) [37] in one of the monomers of M_2-1_ because of the rotational symmetry of its tetrameric arrangement. This binding site is located in a cavity formed by the zinc-finger domain from a monomer of the protein and the core domain from another one. Previously, blind docking calculations were performed for the flavanones following the same procedure applied by Guimarães et al. (2018) for acetylated quercetin derivatives [31], thus identifying the aforementioned binding site and which was later refined by more accurate docking calculations using a reduced conformational search space (70 × 70 × 70 points). Figure S4 shows a comparison for HST and HSD of its lowest energy pose from the largest cluster determined by blind docking and final docking with reduced grid box, where it is possible to see that the orientations of the flavonoid from the blind and final docking calculations were very similar. Figure S4 also presents an overlapping of AMP with flavonoids HST and HSD, indicating that these molecules share the same binding site. The AutoDock 4.2 program [35] was applied to study the binding sites between the flavonoids and the M_2-1_ protein using the Lamarckian Genetic Algorithm (LGA) with number of energy evaluations of 25 million, population size of 250, and RMSD tolerance for cluster analysis of 2 and 5 Å for HST and HSD (due the differences in the number of torsions), respectively. For each docking simulation, 100 different conformers were obtained. The remaining parameters were kept as default. The structural representations were prepared using PyMol software [50] and the maps of non-covalent interactions using PoseView [36] from the Protein*Plus* webserver [51].

### 3.8. Molecular Dynamics

Molecular dynamics (MD) simulations were performed by using GROMACS version 5.0.7 [52]. The hRSV M_2-1_/flavonoid complexes were modeled with the GROMOS54A7 force field [53] and SPC water model [54] was used. The topology parameterizations for the flavonoids HST and HSD were obtained from the ATB server [55]. The initial positions of the polyphenolic compounds for the MD simulations were provided by the molecular docking result from AutoDock 4.2 program [35]. The structural models of the M_2-1_/flavonoid complexes were placed in the center of a 104 Å cubic box solvated by a solution of 150 mN NaCl in water. The protonation status of the amino acid residues of the protein was set based on the results from PROPKA 3.0 [56], considering a pH 7.0. Periodic boundary conditions and NPT ensemble were used in all simulations, maintaining the molecular system at 298 K (25 °C) and 1.0 bar using the modified Berendsen thermostat ($\tau_{T}$ = 0.1 ps) and the Parrinello-Rahman barostat ($\tau_{P}$ = 2.0 ps and compressibility of $4.5\times 10^{-5}\;{\mathrm{bar}}^{-1}$). A cutoff of 12 Å for the Lennard-Jones and Coulomb potentials was used. The particle mesh Ewald (PME) algorithm was used to calculate the long-range electrostatic interactions. For every MD simulation, a time step of 2.0 fs was used and all covalent bonds involving hydrogen atoms were restricted to their equilibrium distances. The conjugate gradient minimization algorithm was applied to relax the atoms in order to avoid overlaps occurring at the beginning of the box construction process. The energy minimization was performed with steepest descent integrator and conjugate gradient algorithm using the maximum force criterion equal to 1000 kJ·mol^−1^·nm^−1^. 50 thousand steps of molecular dynamics were performed for each NVT and NPT equilibration, applying force constants of 1000 kJ·mol^−1^·nm^−2^ to all heavy atoms of the M_2-1_/flavonoid complexes. Lastly, two MD simulations of 20 ns were carried out for data acquisition of the trajectories for the binding model of the HST and HSD to the M_2-1_ protein. As a control calculation, one of the two MD simulations for each flavonoid was extended to 80 ns. After dynamics, the trajectories were concatenated and analyzed according to the following parameters: root mean square deviation (RMSD), percentage occupancy of hydrogen bonds (cutoff distance = 3.5 Å and maximum angle = 30°) obtained from plot_hbmap_generic.pl script [57], number of contacts (<0.6 nm), and distance from the Zn^2+^ ion of the zinc-finger domain of the hRSV M_2-1_ protein to the rigid root of the flavonoids. This rigid root was determined from the AutoDockTools program [49], being that for HST is the C2 carbon atom while for HSD is the first anomeric carbon on the rutinose. The theoretical binding free energy (ΔG*_binding_*) between protein and flavonoids was calculated from the last 10 ns of the 20 ns MD trajectory for each complex using the Molecular Mechanics Poisson-Boltzmann Surface Analysis method implemented in the program g_mmpbsa, along with the MmPbSaStat.py script [38,39]. A bootstrap analysis (*n* = 5000) was performed to obtain standard errors for the energies determined by MM-PBSA analysis. As 20 ns MD simulations were performed in duplicate, the mentioned parameters were also presented as averages. Only hydrogen bonds simultaneously identified in the simulation duplicates were considered for calculating their average values of accumulated percentage occupancy.

## 4. Conclusions

In this study, the experimental techniques of STD-NMR and fluorescence spectroscopy along with computational simulations were used to investigate the binding interactions between the hRSV M_2-1_ protein and the flavanones hesperetin and hesperidin. The STD-NMR experiments unambiguously showed that HST and HSD interacted with the protein, and the binding epitopes mapping revealed that the A and B aromatic rings of these flavanones occupied the binding site in M_2-1_, which was especially true for the HST A-ring, while the HSD rutinose group was exposed. The fluorescence quenching measurements exhibited binding constant (K_b_) values for the M_2-1_/HST complex greater than those for the interaction with HSD, indicating that HST has a higher binding affinity to the protein. The analysis of the thermodynamic parameters for the binding interaction suggests that this process is spontaneous (ΔG < 0) and exothermic (ΔH < 0) for both flavanones, being the M_2-1_/flavonoid complexes stabilized by hydrogen bonds and van der Waals interactions. The computational calculations corroborated with the experimental results, indicating a greater binding affinity of HST (higher magnitude of ΔG*_binding_* from MM-PBSA analysis) to M_2-1_ structure compared to HSD and also revealing that A and B aromatic rings of the flavonoids are closer to the residues inside the protein binding site participating in hydrogen bonds and stacking interactions. The molecular dynamics simulations also showed that the structural models of the molecular complexes for the flavanones interacting in the AMP-binding site of M_2-1_ were stable along all the simulation time, and hydrogen bond established with Lys8 presented a significant percentage occupancy for HST and HSD. Therefore, it can be concluded that HST and HSD bind to hRSV M_2-1_ and their association might impair the biological function of the protein. The results presented herein explain well the findings of plaque reduction assays performed by Kaul and coauthors (1985) [28], which spotted an anti-replicative activity of hesperetin against RSV, noting that M_2-1_ play a role key in the replicative process of this virus.

## Figures and Tables

**Table 1:** Binding constant (K_b_), number of binding sites (*n*), enthalpy change (ΔH), Gibbs free energy change (ΔG), and entropy change (ΔS) of the M_2-1_/flavonoid interaction determined using fluorescence quenching experiments at 288 and 298 K.

Flavonoids	T (K)	K_b_^a^ (× 10^4^ M^−1^)	*n* ^a^	ΔH (kcal.mol^−1^)	ΔG (kcal.mol^−1^)	T·ΔS (kcal.mol^−1^)
HST	288	8.40	1.05	−29.0	−6.5	−22.5
	298	1.53	1.04		−5.7	−23.3
HSD	288	0.96	0.89	−1.7	−5.2	3.5
	298	0.87	0.93		−5.4	3.7

^a^ All correlations coefficients were >0.998 and standard deviations <0.03.

**Table 2:** Theoretical binding free energy (ΔG*_binding_*) determined by MM-PBSA calculations for the structural models of the M_2-1_/flavonoid complexes using the last 10 ns MD trajectories.

Flavonoids	First MD Simulation ΔG*_binding_* (kcal.mol^−1^)	Second MD Simulation ΔG*_binding_* (kcal.mol^−1^)	Average (kcal.mol^−1^)
HST	−40.4 ± 0.8	−30.0 ± 1.0	−35.2
HSD	−27.1 ± 0.4	−29.3 ± 0.5	−28.2

**Table 3:** The accumulated percentage occupancies (%Acc.) of hydrogen bonds formed between M_2-1_ and HST during 20 ns MD trajectory were obtained for the simulation duplicates. The amino acid residues of the protein involved in hydrogen bonds with average values (%Ave.) of accumulated percentage occupancy higher than 10% (highlighted in bold) were considered as a significant non-covalent interaction for the molecular stabilization of the M_2-1_/HST complex.

First MD Simulation					Second MD Simulation					>10%		
Donor ^a^		Acceptor ^a^		%Acc.^b^	Donor ^a^		Acceptor ^a^		%Acc.^b^	Res.^c^	Atm.^d^	%Ave.
K169	NZ	HST	O2,3	0.7	K169	NZ	HST	O2–4	27.036	**K8**	**N**	**70.315**
N174	N	HST	O3,4	11.794	N174	N	HST	O4	2.499	**N174**	**O2**	**69.815**
N174	ND2	HST	O4	0.2	R4	NH1	HST	O3	0.2	**N17**	**ND2**	**26.762**
N5	ND2	HST	O5	7.596	R4	NH2	HST	O3	0.15	**K8**	**NZ**	**25.812**
K8	N	HST	O3–6	44.428	K8	N	HST	O5	96.202	**R20**	**O**	**14.643**
K8	NZ	HST	O3–5	51.174	K8	NZ	HST	O4	0.45	**K169**	**NZ**	**13.868**
F9	N	HST	O3,4	6.447	F9	N	HST	O5	0.1			
N17	ND2	HST	O1,2,6	30.735	N17	ND2	HST	O1,2	22.789			
R20	NH2	HST	O1	0.05	R20	NE	HST	O1,2	25.737			
H22	N	HST	O1,2	1.05	R20	NH1	HST	O1,2	6.947			
H22	NE2	HST	O2	0.15	R20	NH2	HST	O1,2	9.845			
F23	N	HST	O2	0.5	H22	N	HST	O1,2	3.199			
HST	O5	I173	O	0.05	H22	NE2	HST	O3	0.05			
HST	O5	N5	OD1	0.5	HST	O4	N174	O1	1.0			
HST	O5	N5	O	5.547	HST	O4	N174	O2	89.455			
HST	O5	P6	O	0.55	HST	O2	E81	OE1	30.385			
HST	O4	N174	O1	11.894	HST	O2	E81	OE2	9.895			
HST	O4	N174	O2	50.175	HST	O2	N17	OD1	0.5			
HST	O2	N17	OD1	6.797	HST	O2	R20	O	1.949			
HST	O2	N17	O	0.05	HST	O2	H22	ND1	9.345			
HST	O2	G18	O	0.05								
HST	O2	R20	O	27.336								
HST	O2	H22	ND1	3.348								

^a^ The column on the right denotes the amino acid residue and the other on the left, the atom. ^b^ %Acc. is the accumulated percentage occupancy of hydrogen bonds for a type atom of a residue. The details of the percentage occupancy for each type atom of a residue is shown in Table S1. ^c^ Res. denotes an amino acid residue. ^d^ Atm. depicts a type atom of a residue.

**Table 4:** The accumulated percentage occupancies (%Acc.) of hydrogen bonds formed between M_2-1_ and HSD during 20 ns MD trajectory were obtained for the simulation duplicates. The amino acid residues of the protein involved in hydrogen bonds with average values (%Ave.) of accumulated percentage occupancy higher than 10% (highlighted in bold) were considered as a significant non-covalent interaction for the molecular stabilization of the M_2-1_/HSD complex.

First MD Simulation					Second MD Simulation					>10%		
Donor ^a^		Acceptor ^a^		%Acc.^b^	Donor ^a^		Acceptor ^a^		%Acc.^b^	Res.^c^	Atm.^d^	%Ave.
K169	NZ	HSD	O4	0.05	K169	NZ	HSD	O6,8 O14,15	1.75	**R20**	**O**	**22.564**
N174	N	HSD	O4	4.298	N174	N	HSD	O3,4	2.898	**H22**	**ND1**	**17.043**
R4	NE	HSD	O13–15	0.2	N174	ND2	HSD	O3,4	4.098	**K8**	**N**	**14.793**
R4	NH1	HSD	O13–15	1.35	K8	N	HSD	O2	3.998	**F9**	**N**	**10.495**
R4	NH2	HSD	O13–15	0.6	K8	NZ	HSD	O3,4	2.649			
N5	N	HSD	O15	0.1	F9	N	HSD	O2	11.294			
N5	ND2	HSD	O3,4 O13–15	2.699	N17	ND2	HSD	O6,8,9	10.245			
K8	N	HSD	O2,4	25.587	G18	N	HSD	O8,9	0.80			
K8	NZ	HSD	O3,4	1.2	R20	N	HSD	O8	0.05			
F9	N	HSD	O2	9.695	R20	NE	HSD	O8–12	2.1			
N17	ND2	HSD	O5,6,8	1.699	R20	NH1	HSD	O7–12,15	8.946			
R20	NE	HSD	O6,8–10	3.299	R20	NH2	HSD	O7,9 O10,12	2.149			
R20	NH1	HSD	O6,8,9	4.998	H22	N	HSD	O7,11–15	11.094			
R20	NH2	HSD	O6,8–10	0.85	H22	NE2	HSD	O7,11 O12,14,15	4.848			
H22	N	HSD	O7,13	1.1	F23	N	HSD	O5,13,14	0.4			
H22	NE2	HSD	O8,10	0.95	R4	NH2	HSD	O15	0.15			
F23	N	HSD	O5,13,15	2.799	HSD	O5,10 O13–15	H22	ND1	16.193			
HSD	O8,10 O13–15	H22	ND1	17.892	HSD	O13	H22	NE2	0.05			
HSD	O13–15	H22	O	2.899	HSD	O5,8 O10,13,15	R20	O	41.83			
HSD	O15	H22	NE2	0.05	HSD	O8	R20	NH2	0.05			
HSD	O4,14,15	N5	OD1	5.347	HSD	O15	C21	N	0.05			
HSD	O8,13	R20	O	3.248	HSD	O13–15	H22	O	1.5			
HSD	O8	N17	OD1	0.8	HSD	O8–10	E81	OE1	35.382			
HSD	O4	I173	O	0.05	HSD	O8,9	E81	OE2	40.329			
HSD	O4	N174	O1	1.549	HSD	O8,9	N17	OD1	1.649			
HSD	O4	N174	O2	2.849	HSD	O8	G18	O	1.399			
HSD	O4	N5	O	2.299	HSD	O4	K169	O	0.1			
HSD	O4	P6	O	1.949	HSD	O4	T172	OG1	0.05			
					HSD	O4	T172	O	4.598			
					HSD	O4	N174	O1	0.1			
					HSD	O4	N174	O2	3.698			
					HSD	O4	N5	OD1	0.15			
					HSD	O4	P6	O	0.05			

^a^ The column on the right denotes the amino acid residue and the other on the left, the atom. ^b^ %Acc. is the accumulated percentage occupancy of hydrogen bonds for a type atom of a residue. The details of the percentage occupancy for each type atom of a residue is shown in Table S2. ^c^ Res. denotes an amino acid residue. ^d^ Atm. depicts a type atom of a residue.

## Supplementary Materials

Supplementary materials can be found at https://www.mdpi.com/1422-0067/21/6/2241/s1.

## Abbreviations

hRSV	Human Respiratory Syncytial Virus
HST	Hesperetin
HSD	Hesperidin
AMP	Adenosine monophosphate
STD-NMR	Saturation transfer difference by nuclear magnetic resonance
Δ*H*	Enthalpy variation
Δ*S*	Entropy variation
Δ*G*	Gibbs free energy
MD	molecular dynamics
MM-PBSA	Molecular Mechanics Poisson-Boltzmann Surface Area
