# Supplementary material for: Insights into Interactions of Flavanones with Target Human Respiratory Syncytial Virus M2-1 Protein from STD-NMR, Fluorescence Spectroscopy, and Computational Simulations
Source: Int J Mol Sci. 2020 Mar 24;21(6):2241. doi: 10.3390/ijms21062241 (PMC7139479; doi:10.3390/ijms21062241)
Supplement: Supplementary file 1 [file ijms-21-02241-s001.pdf]

Supplementary material

Figure S1 – Analysis of the parameters from duplicates of 20 ns molecular dynamic simulations of the structural models of the complexes formed with HST (A) and HSD (B) determined by AutoDock program. At the top, the RMSDs of non-hydrogen atoms of the flavonoids (black and green line) flavonoids and backbone atoms of the M_{2-1} protein (red and blue line) from the starting structure (AutoDock model) for the complexes are reported as a function of time. In the middle, number of contacts between atoms of M_{2-1} and flavanones for distances < 0.6 nm. At the bottom, distance from Zn^{2+} atom in the zinc-finger domain of the protein to the rigid root of the flavanones.

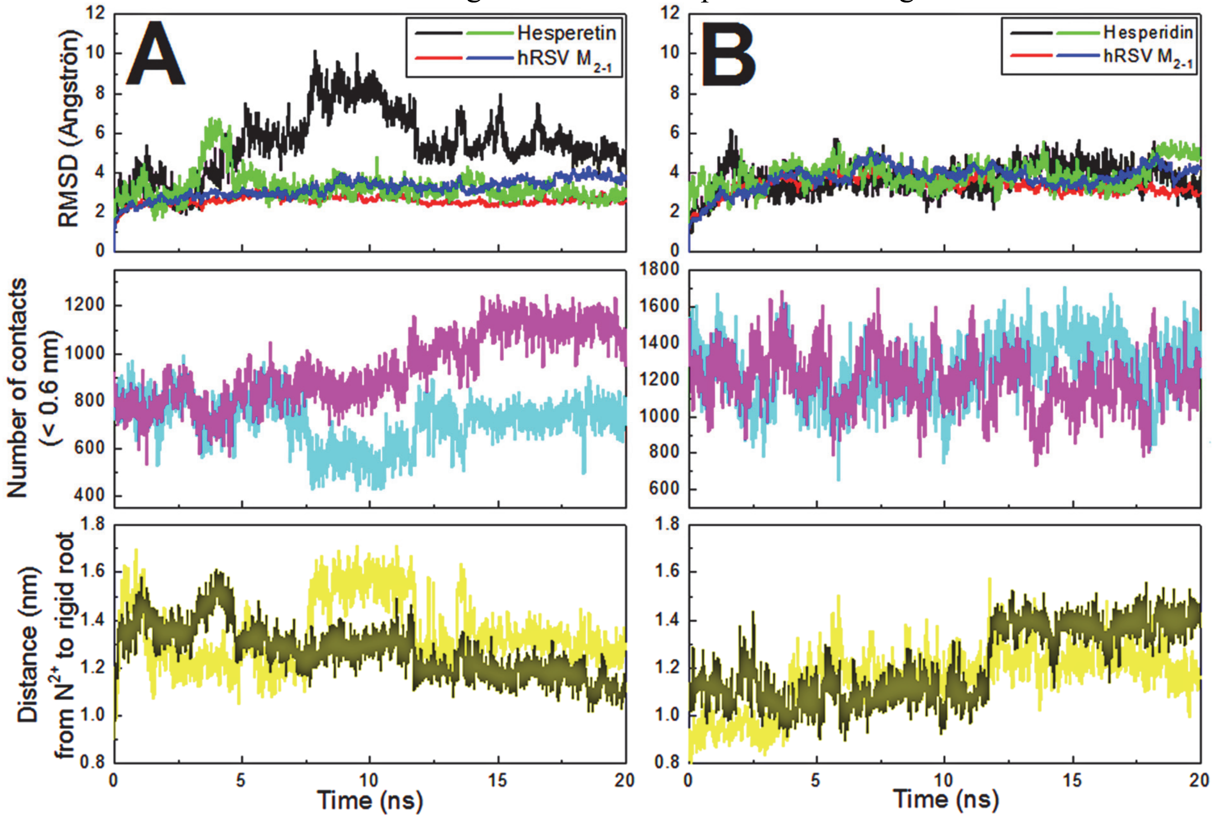

Figure S2 – Comparison of the positions of HST (A) and HSD (B) before and after MD simulations for the structural models of the M2-1/flavonoid complexes. The protein is denoted as a cartoon and the flavonoids as sticks. The green color depicts the position before MD, while cyan and magenta indicate the positions after 20 ns simulations (duplicate). The spheres represent the Zn^{2+} atom in the zinc finger domain of the protein.

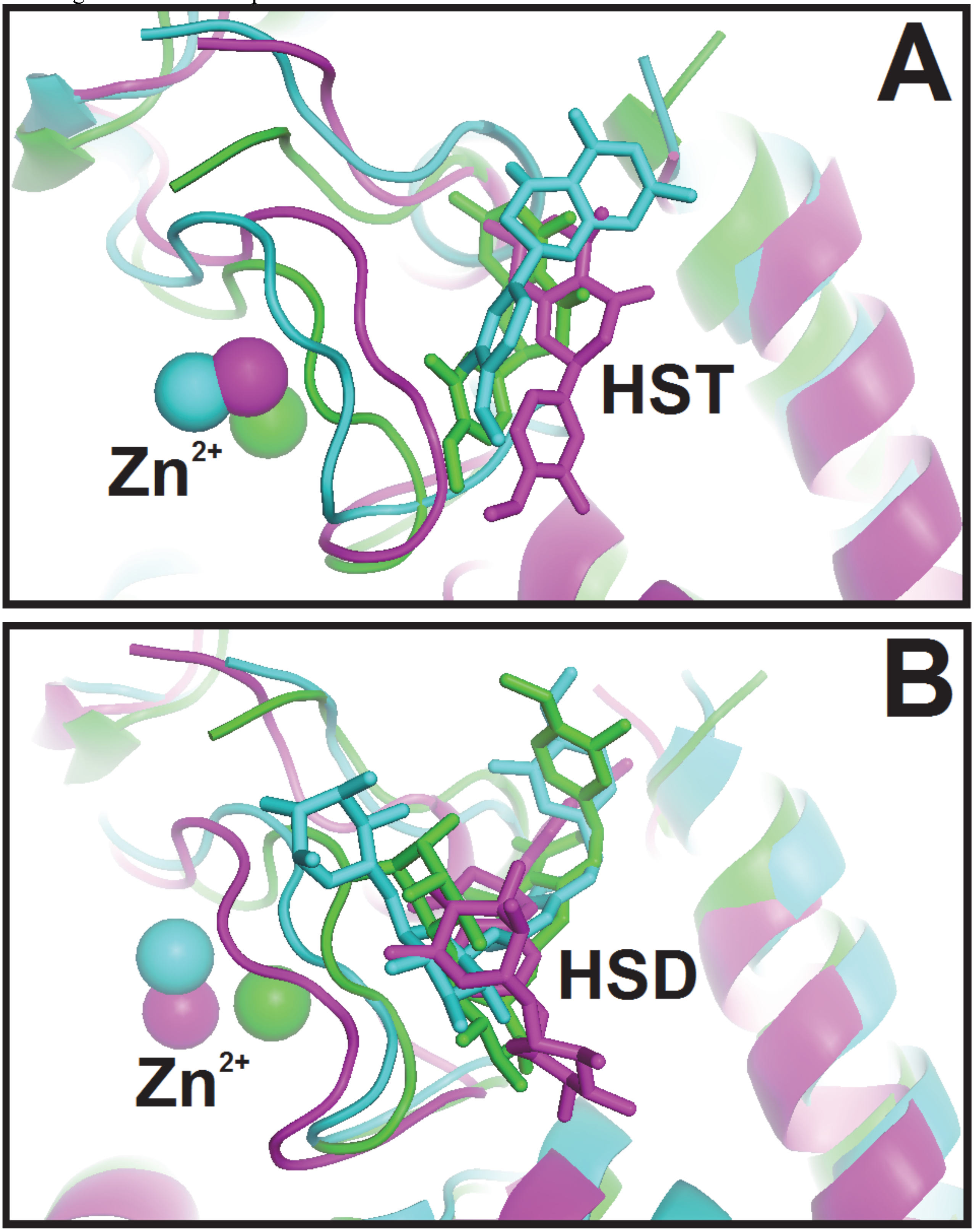

Figure S3 – RMSD values of non-hydrogen atoms of the flavonoids (black line) flavonoids and backbone atoms of the $M_{2\text{-}1}$ protein (red line) calculated from 80 ns MD simulation of the structural models of the complexes formed with HST (A) and HSD (B) determined by AutoDock program.

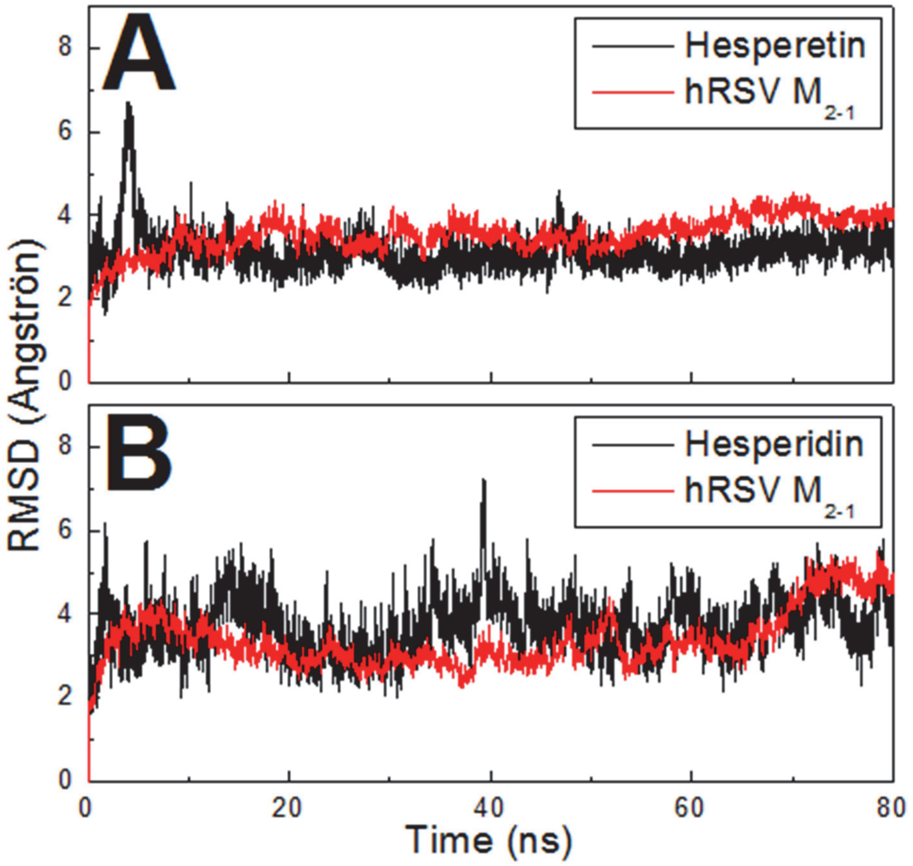

Figure S4: The lowest energy pose from the largest cluster for HST (A) and HSD (B) determined by blind docking (blue) and final docking with reduced grid box (green). The orientation of AMP molecule (yellow) in the binding site of the $M_{2\text{-}1}$ protein was obtained from PDB ID 4CS9 [1]. The protein is represented as a cartoon with the monomers colored in different colors (cyan and gray). The flavonoids and AMP are denoted as sticks and the zinc atom as magenta spheres.

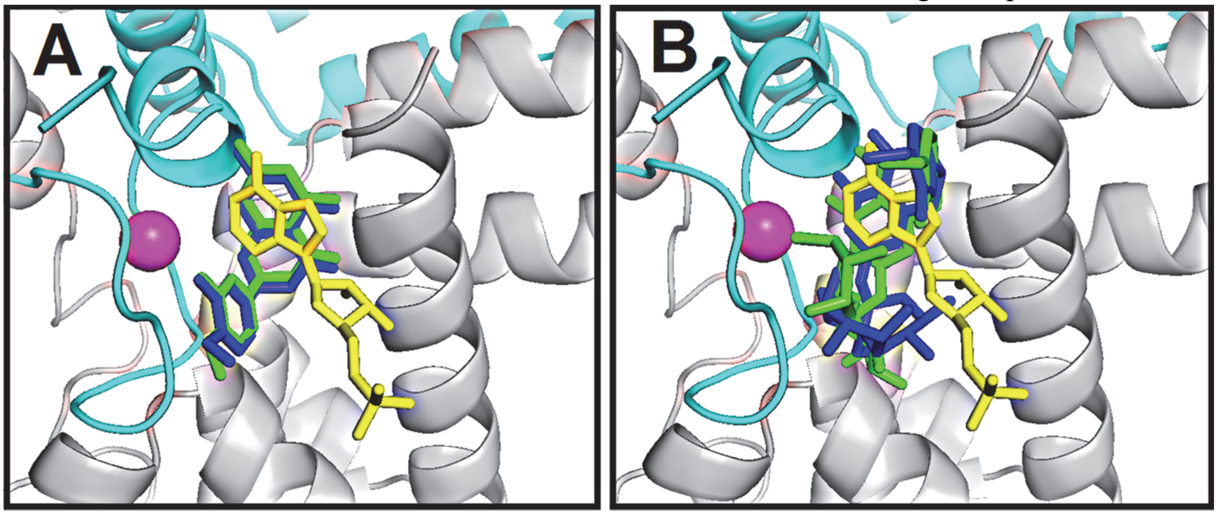

Table S1 – The percentage occupancies (%Occ.) of hydrogen bonds formed between M_{2-1} and HST during 20ns MD trajectory were obtained for the simulation duplicates. The amino acid residues of the protein involved in hydrogen bonds with average values (%Ave.) of accumulated percentage occupancy higher than 10% (bold) were considered as a significant non-covalent interaction for the molecular stabilization of the M_{2-1}/HST complex.

First MD simulation						Second MD simulation						>10%		
Donor[a]		Acceptor[a]		%Occ.	%Acc.[b]	Donor[a]		Acceptor[a]		%Occ.	%Acc.[b]	Res.[c]	Atm.[d]	%Ave.
K169	NZ	HST	O3	0.55	0.7	K169	NZ	HST	O4	1.549	27.036	**K8**	**N**	**70.315**
K169	NZ	HST	O2	0.15		K169	NZ	HST	O3	25.287		**N174**	**O2**	**69.815**
N174	N	HST	O4	3.998	11.794	K169	NZ	HST	O2	0.2		**N17**	**ND2**	**26.762**
N174	N	HST	O3	7.796		N174	N	HST	O4	2.499		**K8**	**NZ**	**25.812**
N174	ND2	HST	O4	0.2		R4	NH1	HST	O3	0.2		**R20**	**O**	**14.643**
N5	ND2	HST	O5	7.596		R4	NH2	HST	O3	0.15		**K169**	**NZ**	**13.868**
K8	N	HST	O5	6.097	44.428	K8	N	HST	O5	96.202				
K8	N	HST	O4	0.25		K8	NZ	HST	O4	0.45				
K8	N	HST	O3	32.484		F9	N	HST	O5	0.1				
K8	N	HST	O6	5.597		N17	ND2	HST	O1	0.15	22.789			
K8	NZ	HST	O5	0.25	51.174	N17	ND2	HST	O2	22.639				
K8	NZ	HST	O4	29.135		R20	NE	HST	O1	3.548	25.737			
K8	NZ	HST	O3	21.789		R20	NE	HST	O2	22.189				
F9	N	HST	O4	0.1	6.447	R20	NH1	HST	O1	0.35	6.947			
F9	N	HST	O3	6.347		R20	NH1	HST	O2	6.597				
N17	ND2	HST	O6	0.4	30.735	R20	NH2	HST	O1	1.099	9.845			
N17	ND2	HST	O1	0.05		R20	NH2	HST	O2	8.746				
N17	ND2	HST	O2	30.285		H22	N	HST	O1	0.45	3.199			
R20	NH2	HST	O1	0.05		H22	N	HST	O2	2.749				
H22	N	HST	O1	0.05	1.05	H22	NE2	HST	O3	0.05				
H22	N	HST	O2	1.0		HST	O4	N174	O1	1.0				
H22	NE2	HST	O2	0.15		HST	O4	N174	O2	89.455				
F23	N	HST	O2	0.5		HST	O2	E81	OE1	30.385				
HST	O5	I173	O	0.05		HST	O2	E81	OE2	9.895				
HST	O5	N5	OD1	0.5		HST	O2	N17	OD1	0.5				
HST	O5	N5	O	5.547		HST	O2	R20	O	1.949				
HST	O5	P6	O	0.55		HST	O2	H22	ND1	9.345				
HST	O4	N174	O1	11.894										
HST	O4	N174	O2	50.175										
HST	O2	N17	OD1	6.797										
HST	O2	N17	O	0.05										

HST	O2	G18	O	0.05
HST	O2	R20	O	27.336
HST	O2	H22	ND1	3.348

[a] The column on the right denotes the amino acid residue and the other on the left, the atom.
[b] %Acc. is the accumulated percentage occupancy of hydrogen bonds for a type atom of a residue.
[c] Res. denotes an amino acid residue.
[d] Atm. depicts a type atom of a residue.

Table S2 – The percentage occupancies (%Occ.) of hydrogen bonds formed between M_{2-1} and HSD during 20 ns MD trajectory were obtained for the simulation duplicates. The amino acid residues of the protein involved in hydrogen bonds with average values (%Ave.) of accumulated percentage occupancy higher than 10% (bold) were considered as a significant non-covalent interaction for the molecular stabilization of the M_{2-1}/HSD complex.

First MD simulation						Second MD simulation						>10%		
Donor[a]		Acceptor[a]		%Occ.	%Acc.[b]	Donor[a]		Acceptor[a]		%Occ.	%Acc.[b]	Res.[c]	Atm.[d]	%Ave.
K169	NZ	HSD	O4	0.05		K169	NZ	HSD	O15	0.25		**R20**	**O**	**22.564**
N174	N	HSD	O4	4.298		K169	NZ	HSD	O14	0.5	1.75	**H22**	**ND1**	**17.043**
R4	NE	HSD	O15	0.05		K169	NZ	HSD	O8	1.0		**K8**	**N**	**14.793**
R4	NE	HSD	O14	0.1	0.2	K169	NZ	HSD	O6	0.05		**F9**	**N**	**10.495**
R4	NE	HSD	O13	0.05		N174	N	HSD	O4	1.699	2.898			
R4	NH1	HSD	O15	0.15		N174	N	HSD	O3	1.199				
R4	NH1	HSD	O14	0.85	1.35	N174	ND2	HSD	O4	2.149	4.098			
R4	NH1	HSD	O13	0.35		N174	ND2	HSD	O3	1.949				
R4	NH2	HSD	O15	0.05		K8	N	HSD	O2	3.998				
R4	NH2	HSD	O14	0.45	0.6	K8	NZ	HSD	O4	1.749	2.649			
R4	NH2	HSD	O13	0.1		K8	NZ	HSD	O3	0.9				
N5	N	HSD	O15	0.1		F9	N	HSD	O2	11.294				
N5	ND2	HSD	O15	0.2		N17	ND2	HSD	O9	1.199				
N5	ND2	HSD	O14	0.3	2.699	N17	ND2	HSD	O8	8.796	10.245			
N5	ND2	HSD	O13	0.05		N17	ND2	HSD	O6	0.25				

N5	ND2	HSD	O4	0.7		G18	N	HSD	O9	0.3	0.8
N5	ND2	HSD	O3	1.449		G18	N	HSD	O8	0.5	
K8	N	HSD	O2	24.338	25.587	R20	N	HSD	O8	0.05	
K8	N	HSD	O4	1.249		R20	NE	HSD	O12	0.75	
K8	NZ	HSD	O4	0.55	1.2	R20	NE	HSD	O11	0.35	
K8	NZ	HSD	O3	0.65		R20	NE	HSD	O10	0.6	2.1
F9	N	HSD	O2	9.695		R20	NE	HSD	O9	0.35	
N17	ND2	HSD	O8	1.649		R20	NE	HSD	O8	0.05	
N17	ND2	HSD	O6	0.05	1.699	R20	NH1	HSD	O15	0.05	
N17	ND2	HSD	O5	0.1		R20	NH1	HSD	O12	2.399	
R20	NE	HSD	O10	0.05		R20	NH1	HSD	O11	3.248	
R20	NE	HSD	O9	0.4	3.299	R20	NH1	HSD	O10	4.698	8.946
R20	NE	HSD	O8	2.799		R20	NH1	HSD	O9	0.4	
R20	NE	HSD	O6	0.05		R20	NH1	HSD	O8	2.799	
R20	NH1	HSD	O9	0.8		R20	NH1	HSD	O7	0.05	
R20	NH1	HSD	O8	3.698	4.998	R20	NH2	HSD	O12	0.6	
R20	NH1	HSD	O6	0.5		R20	NH2	HSD	O10	1.049	2.149
R20	NH2	HSD	O10	0.05		R20	NH2	HSD	O9	0.4	
R20	NH2	HSD	O9	0.45	0.85	R20	NH2	HSD	O7	0.1	
R20	NH2	HSD	O8	0.2		H22	N	HSD	O15	5.547	
R20	NH2	HSD	O6	0.15		H22	N	HSD	O14	1.449	11.094
H22	N	HSD	O13	0.75	1.1	H22	N	HSD	O13	2.099	
H22	N	HSD	O7	0.35		H22	N	HSD	O12	0.35	
H22	NE2	HSD	O10	0.85		H22	N	HSD	O11	0.25	
H22	NE2	HSD	O8	0.1	1.0	H22	N	HSD	O7	1,399	
HSD	O15	H22	NE2	0.05		H22	NE2	HSD	O15	0,95	
F23	N	HSD	O15	0.05		H22	NE2	HSD	O14	0,45	
F23	N	HSD	O13	0.35	2.799	H22	NE2	HSD	O12	1.399	
F23	N	HSD	O5	2.399		H22	NE2	HSD	O11	1.999	4.898
HSD	O15	H22	ND1	0.3		H22	NE2	HSD	O7	0.05	
HSD	O14	H22	ND1	0.15		HSD	O13	H22	NE2	0.05	
HSD	O13	H22	ND1	0.65	17.892	F23	N	HSD	O14	0.15	
HSD	O10	H22	ND1	15.942		F23	N	HSD	O13	0.05	0.4
HSD	O8	H22	ND1	0.85		F23	N	HSD	O5	0.2	
HSD	O15	H22	O	1.899		R4	NH2	HSD	O15	0.15	
HSD	O14	H22	O	0.2	2.899	HSD	O15	H22	ND1	10.545	16.193
HSD	O13	H22	O	0.8		HSD	O14	H22	ND1	2.749	
HSD	O15	N5	OD	0.05	5.347	HSD	O13	H22	ND	2.149	

			1						1		
HSD	O14	N5	OD1	0.1		HSD	O10	H22	ND1	0.15	
HSD	O4	N5	OD1	5.197		HSD	O5	H22	ND1	0.6	
HSD	O13	R20	O	0.15	3.248	HSD	O15	R20	O	0.1	
HSD	O8	R20	O	3.098		HSD	O13	R20	O	0.3	
HSD	O8	N17	OD1	0.8		HSD	O10	R20	O	0.15	41.83
HSD	O4	I173	O	0.05		HSD	O8	R20	O	6.647	
HSD	O4	N174	O1	1.549		HSD	O5	R20	O	34.633	
HSD	O4	N174	O2	2.849		HSD	O8	R20	NH2	0.05	
HSD	O4	N5	O	2.299		HSD	O15	C21	N	0.05	
HSD	O4	P6	O	1.949		HSD	O15	H22	O	0.6	
						HSD	O14	H22	O	0.55	1.5
						HSD	O13	H22	O	0.35	
						HSD	O10	E81	OE1	0.05	
						HSD	O9	E81	OE1	17.191	35.382
						HSD	O8	E81	OE1	18.141	
						HSD	O9	E81	OE2	19.29	40.329
						HSD	O8	E81	OE2	21.039	
						HSD	O9	N17	OD1	0.25	1.649
						HSD	O8	N17	OD1	1.399	
						HSD	O8	G18	O	1.399	
						HSD	O4	K169	O	0.1	
						HSD	O4	T172	OG1	0.05	
						HSD	O4	T172	O	4.598	
						HSD	O4	N174	O1	0.1	
						HSD	O4	N174	O2	3.698	
						HSD	O4	N5	OD1	0.15	
						HSD	O4	P6	O	0.05	

[a] The column on the right denotes the amino acid residue and the other on the left, the atom.
[b] %Acc. is the accumulated percentage occupancy of hydrogen bonds for a type atom of a residue.
[c] Res. denotes an amino acid residue.
[d] Atm. depicts a type atom of a residue.

References

[1] Leyrat, C.; Renner, M.; Harlos, K.; Huiskonen, J.T.; Grimes, J.M. Drastic changes in conformational dynamics of the antiterminator M2-1 regulate transcription efficiency in *Pneumovirinae*. *eLIFE* **2014**, e02674.
